# Effects of Dried Olive Pulp Dietary Supplementation on Quality Characteristics and Antioxidant Capacity of Pig Meat

**DOI:** 10.3390/foods9010081

**Published:** 2020-01-11

**Authors:** Anna Tsala, Vasilios Mpekelis, Giannis Karvelis, Panagiotis Tsikakis, Michael Goliomytis, Panagiotis Simitzis

**Affiliations:** 1 Laboratory of Animal Breeding and Husbandry, Department of Animal Production, Agricultural University of Athens, Iera Odos 75, 11855 Athens, Greece; stud213078@aua.gr (A.T.); mgolio@aua.gr (M.G.); 2 Sparta Life S.A., Sparti-Gytheio National Road, 23100 Sparta, Greece; info@spartalife.gr; 3 Nuevo S.A., Schimatari, 32009 Viotia, Greece; 4 Tsikakis–Giannopoulos S.A., Lefki Anogion, 23100 Sparta, Greece; p.tsikakis@gmail.com

**Keywords:** dried olive pulp, pork, meat quality, meat oxidative stability, agro-industrial by-products, sustainability

## Abstract

Olive pulp belongs to agro-industrial by-products, and its addition into livestock diets generally result in neutral or positive effects on performance. However, the data concerning the effects of olive by-products on pork meat characteristics are scarce. The aim of this preliminary study was therefore to examine the effects of dried olive pulp (DOP) dietary supplementation on quality parameters and oxidative stability of pig meat. Twenty finishing pigs were allocated to two groups: C that was provided with a control diet, and DOP that was fed with an isonitrogenous and isoenergetic diet supplemented with dried olive pulp at the level of 50 g/kg or 5%. As indicated, cold carcass weight, pH, lightness, redness, cooking loss, and tenderness were not influenced by DOP inclusion. Only meat yellowness (a*) was significantly decreased in DOP meat samples. Moreover, meat oxidation values tended to decrease in the DOP group after one day, but no further differences were observed after four, six, and eight days of refrigerated storage. It can be concluded that DOP dietary supplementation could be recommended as a feasible approach, especially in the Mediterranean region to reduce diet formulation costs, while no significant side effects on pork meat quality are observed.

## 1. Introduction

Olive (*Olea europaea*) cultivation plays an important economic and social role in the Mediterranean region. In 2014 more than 1.74, 0.29, and 0.21 million tons of olive oil were produced in Spain, Italy, and Greece, respectively [1]. It is concluded that olive oil extraction produces a high quantity of olive by-products (pulp or cake), which represents a severe environmental problem [2]. Therefore, utilization of these residues in the diets of farm animals as a complementary energy source, due to their high residual oil content, might reduce the environmental pollution and costs related to waste management and animal feeding [3]. However, the composition of olive by-products is influenced by the cultivation conditions (geographic origin, year, etc.) and the method of oil extraction, and further determines its nutritional value (oil content, crude protein, and fiber) and preservation characteristics [3].

Previous studies have already pointed out that it is feasible to include olive cake in the diets of ruminants with neutral or positive effects on performance [4]. In dairy ewes, the inclusion of partly-destoned olive cake at the level of 20% resulted in an increase of milk yield and unsaturated/saturated fatty acid ratios, without negative effects on milk chemical composition and clotting properties. This contributed to an improvement of its nutritional characteristics and a decrease of the atherogenic and thrombogenic indices [5]. Moreover, olive cake incorporated up to the level of 12% of the basal diet could also ensure a normal growth performance in lambs [6]. The addition of a similar level of olive cake (15%) as a replacer of the concentrate had no significant effects on daily gain, carcass weight, and dressing percentage of lambs [7].

Apart from ruminants, inclusion of olive pulp up to 9% into the diets of laying hens does not affect productive performance [8]. Moreover, dietary supplementation with olive cake at the level of 57 g/kg reduces the levels of cholesterol and total saturated fatty acids and increases that of total monounsaturated fatty acids (MUFA), total polyunsaturated fatty acids (PUFA), total n-3 PUFA, and docosahexanoic acid in egg yolk [9]. In finishing pigs, feed intake, body gain, carcass weight, and meat MUFA levels were increased and fat depth and meat saturated fatty acids (SFA) content were decreased after the inclusion of olive cake up to 100 g/kg diet [10]. An increase of MUFA concentration in meat of pigs fed partially defatted olive cake supplemented diets (120 g/kg) was also observed without other side effects on growth performance, carcass quality, and microbial counts [11]. Inclusion of olive by-products into the diet of other farm animals also increases MUFA levels (especially oleic acid) of the derived products, i.e., rabbit meat [12], broiler meat [13], lamb meat [14], and ewe milk [15].

As indicated by the existing literature, there are scarce data concerning the effects of the dietary inclusion of olive by-products on pig meat quality. The present study was therefore aimed at examining the impact of dried olive pulp dietary supplementation on pH, color parameters, cooking loss, tenderness, and oxidative stability of pig meat.

## 2. Materials and Methods

### 2.1. Animals and Treatments

This preliminary study was conducted in a commercial farm localized in Sparti, Greece. Twenty castrated male crossbred pigs (PIC 337 × (PIC Landrace × PIC Large White)) were the animals used. Pigs were 4.5 months old at the beginning of the experiment and they were randomly assigned according to their body weight to the two dietary treatment groups (C and DOP) with equal mean liveweights. Pigs of each dietary treatment group were kept together in identical pens, with the same direction and orientation, equipped with similar troughs for feeding and had free access to food and water. During the experimental period, which lasted 30 days, the first group was fed the control diet and the other group consumed the DOP dietary supplemented diet that contained dried olive pulp at the level of 50 g/kg. The chemical analysis of DOP used in the present study is described in detail by Papadomichelakis et al. [12]. In brief, its dry matter (DM) is 945 g/kg and DOP contains 85.7 g crude protein, 174.6 g crude fat, 276.0 g crude fiber, and 61.4 g ash per kg DM. Neutral detergent fiber (NDF) and acid detergent fiber (ADF) levels of DOP were 48.68% and 40.74%, respectively. The presence of hydroxytyrosol and oleuropein was confirmed in the DOP at the levels of 229 mg/kg and 1007 mg/kg DM, respectively. Pig diets were formulated to meet the nutrient requirements of piglets [16] and were both isonitrogenous and isoenergetic. The composition and analysis of the experimental diets are shown in Table 1.

### 2.2. Carcass and Meat Quality Measurements

At the age of 165 ± 4 days, pigs were fasted for 12 h and slaughtered. Slaughter processing was conducted at a commercial abattoir after 30 days of feeding with the experimental diets. Cold carcass weight was recorded after an overnight chilling at 24 h postmortem. At the same time, the *Longissimus thoracis* muscle was removed from the carcass and the section between the last rib and the interface between the 12th and 13th ribs was used for further quality assessment.

pH was measured using a pHM210 MeterLab pH system (Copenhagen, Denmark) with the electrode inserted into the center of the *Longissimus thoracis* muscle at the last rib, 24 h after slaughter. The pH meter was calibrated in buffers at pH 4.0 and 7.0 (Merck, Darmstadt, Germany) at ambient temperature. The part of the muscle between the 12th and 13th ribs was sliced across the fibers, left exposed to the air for 30 min, and meat color was measured (3 measurements per sample) at room temperature (~20 °C) on the cut surface using a Miniscan XE (HunterLab, Reston, VA, USA) chromameter set on the L*, a*, and b* systems (CIE 1976, Commission International de l’ Eclairage). A white and a black tile were used as standards. Samples (80 ± 2 g, 2 cm thickness) of *Longissimus thoracis* muscle were placed in plastic bags and cooked in a water bath at 80 °C for 50 min (internal temperature 72 ± 1 °C), left under running water for 30 min, and then placed in a refrigerator at about 4 °C for 24 h. The meat sample was weighed again to estimate the percentage of cooking loss (%). Five sub samples with a cross section of 1 cm^2^ were cut parallel to the muscle fibers and the shear force value of the *Longissimus thoracis* muscle was measured using a Warner Bratzler (WB) shear blade fitted to a Zwick Testing Machine Model Z2.5/TN1S (Zwick GmbH & Co., Ulm, Germany). Peak force values in Newton were recorded.

Lipid oxidation was assessed on the basis of the malondialdehyde (MDA) formed during storage. In the present study, MDA contents in *Longissimus thoracis* muscle samples were determined 1, 4, 6, and 8 days after refrigerated storage (4 °C) by applying a selective third-order derivative spectrophotometric method [17]. Derivative instead of conventional spectrophotometry was preferred as improved sensitivity, specificity, and reliability of the measurements are observed. Briefly, 2 g of each meat sample (2 samples per pig) were homogenized (Edmund Buehler 7400 Tuebingen/H04, Tübingen, Germany) in the presence of 5 mL butylated hydroxytoluene (BHT) in hexane (8 g/L) and 8 mL aqueous trichloroacetic acid (TCA) (50 g/L), and the mixture was centrifuged for 3 min at 3000× *g*. The top hexane layer was removed and a 2.5 mL aliquot from the bottom layer was stirred with 1.5 mL aqueous 2-thiobarbituric acid (TBA) (8 g/L) to be further incubated at 70 °C for 30 min. The mixture was then cooled under tap water and subjected to third-order derivative (3D) spectrophotometry (Hitachi U3010 Spectrophotometer) in the range of 500–550 nm. MDA levels (ng/g wet tissue) in analyzed samples were estimated on the basis of the height of the third-order derivative peak at 521.5 nm, by referring to slope and intercept data of the computed least-squares fit of the standard calibration curve prepared using 1,1,3,3-tetraethoxypropane (TEP), the MDA precursor.

### 2.3. Statistical Analysis

Cold carcass weight and meat quality characteristics, such as pH 24, color parameters (L*, a*, and b*), cooking loss (%), and shear force value (N) measurements were subjected to analysis of variance with the nutritional treatment as fixed effect. Malondialdehyde (MDA) concentration was analyzed using a mixed model appropriate for repeated measurements per subject, which included the effects of nutritional treatment as fixed effect. The level of significance was set at 0.05 and results are presented as least squares (LS) mean ± standard error of mean (S.E.M.). All model analyses were performed by the statistical analysis software Sas/Stat [18].

## 3. Results

No significant differences were observed in cold carcass weight between the two experimental groups (*p* > 0.05). pH and color values were also not significantly influenced by dietary DOP inclusion (*p* > 0.05), apart from yellowness (a*) that had a decreased value in DOP meat samples (*p* < 0.05). Moreover, incorporation of DOP at the level of 5% did not result in different values for cooking loss and shear force (Table 2).

As indicated in Table 3, MDA values tended to be lower in the DOP compared to the control group after one day of refrigerated storage (*p* = 0.053). However, this reduction in DOP meat samples did not remain after four, six, and eight days of refrigerated storage, since the MDA values were not different between the two experimental groups (*p* > 0.05). In general, refrigerated storage increased lipid oxidation values, as shown by the greater MDA values with time of storage (*p* < 0.01).

## 4. Discussion

As indicated, no effect of DOP dietary supplementation on cold carcass weight was observed in the present preliminary study. Joven et al. [10] reached the same conclusion after the inclusion of olive cake at the level of 50 g/kg in the diets of finishing pigs. No effect on carcass weight of pigs fed partially defatted olive cake (PDOC) supplemented diets (120 g/kg) was also observed [11]. On the other hand, Garcia-Casco et al. [19] found a significant decrease in the carcass yield of pigs fed with dried olive pulp supplemented diets, although premium cut yields were not influenced. The exact reasons for the discrepancies shown in the literature are speculative but they could be attributed to the variability of olive by-products chemical composition (different oil extraction methods), to differences in the content of the major bioactive compounds among the different olive varieties, in the levels and period of supplementation and in the pig genetic origin (hybrid).

As it can be concluded by the results of the present study, incorporation of DOP into the diets of finishing pigs did not significantly affect the examined meat quality parameters apart from yellowness. Meat acidity (pH), lightness, redness, cooking loss, and tenderness were not influenced by DOP inclusion into the pig diets. Ferrer et al. [11] also observed no differences in meat pH and color parameters of pigs fed PDOC supplemented diets. In addition, water holding capacity of pork was also not influenced by olive cake dietary supplementation [10]. On the other hand, meat lightness linearly decreased and yellowness tended to linearly decrease as olive cake levels increased in the diet of pigs (from 50 to 150 g/kg) [9]. A possible explanation for the decreased value of meat yellowness (a*) observed both in the present study and in literature is the dark color of the DOP that could influence coloration of pork meat. Moreover, dietary supplementation of rabbits with olive pomace [12] and of broilers with polyphenols extracted from olive oil industry waste [20] did not affect values of meat pH, cooking loss, tenderness, and color parameters.

Meat oxidation stability assessed by MDA values tended to be improved in the DOP group after one day of refrigerated storage. However, no further differences were observed after four, six and eight days of storage. The results of previous studies regarding the effects of olive oil by-product dietary supplementation on meat oxidation values are controversial. Decreased oxidation rates in beef [21] and lamb meat [14] were observed after the dietary supplementation with olive by-products, since they contain several substances with confirmed antioxidant and radical scavenging activity, such as oleuropein, hydroxytyrosol (3,4-DHPEA), tyrosol (p-HPEA), and their secoiridoid derivatives (dialdheydic form of decarboxymethyl elenolic acid, 3,4-DHPEA-EDA or p-HPEA-EDA) [22]. On the other hand, reduced oxidative stability was observed after the inclusion of olive by-products into the diet of broilers [13] and rabbits [12]. These discrepancies indicate that many factors could influence the antioxidant potential of olive by-products, including peroxide value and polyphenol levels. Moreover, the level of DOP dietary supplementation may disturb the equilibrium between pro- and antioxidative meat content due to its high auto-oxidation rates. A delicate balance between antioxidants and prooxidants in cells is an important determinant for various physiological processes and several authors have already suggested that high inclusion rates of natural antioxidants could lead to pro-oxidant effects [23,24]. However, further research is warranted to elucidate the exact mechanisms underlying this balance in pork meat.

## 5. Conclusions

The results of the present preliminary study clearly demonstrated that the inclusion of olive cake into the pig diets at the level of 50 g/kg could be proposed as an advantageous strategy, especially in Mediterranean areas, allowing exploitation of an important agro-industrial by-product to reduce production costs for pig feeding while maintaining the high quality of pork meat products.

## Figures and Tables

**Table 1:** Ingredients and analysis of the experimental diets.

**Ingredients (g/kg)**	**Diet ^1^**	
	**C**	**DOP**
Maize	160.5	144.2
Soft wheat	200	200
Barley	225	225
Wheat bran	75	30
Soybean meal (440 g CP/kg)	155	163
Soybean oil	7.5	10
Bakery meal	150	150
Dried olive pulp	0	50
Limestone	11.5	11.5
Sodium chloride	3	3
Monocalcium phosphate	0	0.8
TS FIN 991 PIG Compound Feed ^2^	12.5	12.5
**Calculated chemical composition (g/kg)**		
Dry matter	881.2	885.5
Crude protein	159.7	159.2
Ether extract	31.6	39.8
Crude fibre	43.6	51.7
NDF ^3^	147.9	152.0
ADF ^4^	53.2	68.1
Starch	391.8	389.8
Sugar	40.94	38.98
Ash	49.19	50.88
Digestible energy (MJ/kg)	13.81	13.86
Calcium	8.07	8.16
Available phosphorus	2.93	2.93
Sodium	1.81	1.87
Methionine + cystine	5.52	5.30
Lysine	9.05	8.99
Threonine	6.04	6.10
Valine	7.42	7.44

^1^ C, control diet; DOP, dried olive pulp dietary supplemented diet (50 g/kg). ^2^ TS FIN 991 PIG Compound Feed (Nuevo S.A., Schimatari, Greece) provided per kg of diet: 312,000 IU vitamin A (retinyl acetate), 96,000 IU vitamin D3 (cholecalciferol), 16,000 mg vitamin E (dl-α-tocopheryl acetate), 20,000 mg betaine, 60 mg I, 20 mg Se, 8000 mg Fe, 2800 mg Mn, 1200 mg Cu and 6000 mg Zn, 80,000 mg l-lysine, 175,000 mg l-lysine sulphate, 36,000 mg l-threonine. ^3^ Neutral detergent fiber. ^4^ Acid detergent fiber.

**Table 2:** Effects of dietary dried olive pulp (DOP) supplementation on quality characteristics of *Longissimus thoracis* muscle in pigs (least squares (LS) means ± standard error of the mean (S.E.M.)).

Parameter	Diet ^1^		S.E.M.	*p*-Value
	C	DOP		
Cold Carcass Weight (kg)	76.16	76.63	0.80	0.682
pH	5.86	5.92	0.04	0.275
L*	51.32	49.63	0.76	0.131
Color^2^ a*	10.85	10.74	0.29	0.794
b*	16.12 ^a^	15.20 ^b^	0.28	0.033
Cooking loss (%)	32.12	32.55	0.39	0.449
Shear Force (N)	22.11	20.39	1.58	0.452

^1^ C, control diet; DOP, dried olive pulp dietary supplemented diet (50 g/kg). ^2^ L*, lightness; α*, redness; b*, yellowness. ^a^,^b^ Means within a row with different letters are significantly different (*p* < 0.05).

**Table 3:** Effects of dietary dried olive pulp (DOP) supplementation and duration of refrigerated storage on lipid oxidation (malondialdehyde (MDA), ng/g) of *Longissimus thoracis* muscle in pigs (LS means ± S.E.M.) (higher levels of MDA indicate higher rates of lipid oxidation).

Storage Period (Days, at 4 °C)	Diet *		S.E.M.	*p*-Value
	C	DOP		
1	32.48 ^a^	28.87 ^b^	1.23	0.053
4	46.45	47.92	3.30	0.755
6	62.73	56.40	4.16	0.296
8	67.46	67.32	5.32	0.985

* C, control diet; DOP, dried olive pulp dietary supplemented diet (50 g/kg). ^a^,^b^ Means within a row with different letters tend to be different.
